# An outbreak of cholera in western Kenya, 2015: a case control study

**DOI:** 10.11604/pamj.supp.2017.28.1.9477

**Published:** 2017-11-06

**Authors:** Elvis O. Oyugi, Waqo Boru, Mark Obonyo, Jane Githuku, Dickens Onyango, Alfred Wandeba, Eunice Omesa, Tabitha Mwangi, Hudson Kigen, Joshua Muiruri, Zeinab Gura

**Affiliations:** 1 Field Epidemiology and Laboratory Training Program, Ministry of Health, Kenya,; 2 Ministry of Agriculture, Livestock and Fisheries, Kenya,; 3 Kisumu County Department of Health, Ministry of Health, Kenya

**Keywords:** Cholera, outbreak, independent factors, Kenya

## Abstract

**Introduction:**

in February 2015, an outbreak of acute watery diarrhea was reported in two sub counties in western Kenya. *Vibrio cholera*e 01 serotype Ogawa was isolated from 26 cases and from water samples collected from a river mainly used by residents of the two sub-counties for domestic purposes. We carried out an investigation to determine factors associated with the outbreak.

**Methods:**

we conducted a frequency matched case control study in the community. We defined cases as episodes of watery diarrhea (at least three motions in 24 hours) in persons ≥ 2 years who were residents of Rongo or Ndhiwa sub-counties from January 23-February 25, 2015. Cases were systematically recruited from a cholera line list and matched to two controls (persons without diarrhea since January 23, 2015) by age category and residence. A structured questionnaire was administered to evaluate exposures in cases and controls and multivariable logistic regression done to determine independent factors associated with the outbreak.

**Results:**

we recruited 52 cases and 104 controls. Females constituted 61% (95/156) of all participants. Overall latrine coverage was 58% (90/156). Latrine coverage was 44% (23/52) for cases and 64% (67/104) for controls. Having no latrine at home (aOR = 10.9; 95% CI: 3.02-39.21), practicing communal hand washing in a basin (aOR = 6.5; 95% CI: 2.30-18.11) and vending of food as an occupation (aOR = 3.4; 95% CI: 1.06-10.74) were independently associated with the outbreak.

**Conclusion:**

poor latrine coverage and personal hygiene practices were identified as the main drivers of the outbreak. We recommended improved public health education on latrine usage and promotion of hand washing with soap and water in the community.

## Introduction

Cholera is a diarrheal disease caused by the ingestion of bacterium *Vibrio cholera* in faecally contaminated water or food, and it presents with a sudden onset of acute watery diarrhea leading to severe dehydration that can be fatal [1]. It remains a challenge in poor countries with improper sanitation, inadequate water supply, unprotected water sources and crowded or displaced populations [2-6]. Globally, there is still a high burden of cholera, especially in developing countries and it is estimated that approximately 1.4 billion people are at risk in endemic countries, with disease incidence greatest in children aged below five years [7]. Most outbreaks have taken place in Africa, a region that has accounted for 46% of all cases reported between 1970 and 2011 and has recorded high case fatality rates in the past [8-10].

In Kenya, multiple outbreaks of cholera have been reported since 1971 [11], including five distinct outbreaks reported in 2005 [12]. Factors that have been noted to contribute to these outbreaks include the interaction between climatic, environmental and demographic factors, with increased risk in areas that border large water bodies, have shortage of health facilities or experience changes in rainfall trends [13,14]. During outbreaks, water treatment using chlorine and provision of clean piped water to the populace, and proper handwashing are key measures of intervention [3, 15]. In Western Kenya, Migori county experienced previous cholera outbreak in the year 2010 that had a high case fatality rate (CFR) of 9% and one of the contributory factors to this high CFR was the delay in laboratory confirmation of the suspected cases at the beginning of the outbreak [16]. On February 5, 2015, the Ministry of Health (MOH) was notified of an increase in the number of patients presenting with acute watery diarrhea in two counties located in Western Kenya, including four deaths. *Vibrio cholera*e 01 had been isolated from a water sample taken from a river that was used by the residents for their domestic water supply. A team from the Field Epidemiology and Laboratory Training Program (FELTP) investigated the outbreak between February 16 and 25, 2015. Our objectives were to determine the magnitude of the outbreak in the two counties, characterize the cholera cases in terms of time, place and person and identify risk factors for the outbreak.

## Methods

### Retrospective review of cholera outbreak line list

We reviewed and updated the outbreak line list from January 30, 2015 up to March 2, 2015. The variables recorded in the line list were: name, age, sex, residence, date of onset of diarrhea, date of treatment or admission to health facility and the outcome status. For entry into the line list, we defined a suspected cholera case as acute onset of watery diarrhea (at least 3 motions in 24 hours) in a person ≥ 2 years, who was a resident of Migori or Homa Bay counties between January 23, 2015 and March 2, 2015. A probable case was defined as having contact with a confirmed case and a confirmed case was defined as the laboratory isolation of *Vibrio cholera*e serotypes 01 or 0139 in stool culture from a person meeting the probable or suspect case definitions.

### Hypothesis generation

We conducted informal interviews with the County Health Management Team (CHMT) from Migori and Homa Bay and noted that the average toilet coverage in the two counties was approximately 60%. They also reported that river Riana which was used by residents of both counties for domestic water supply was prone to pollution from burst sewerage system upstream in neighboring Kisii county. Based on a preliminary review of the outbreak line list, we identified that most cases were clustered along administrative locations bordering river Riana, especially in Rongo and Ndhiwa sub counties located in Migori and Homa Bay counties respectively. *Vibrio cholera*e, which was confirmed as the cause of the outbreak, had been isolated in a water sample from this river. We therefore hypothesized that the river water could have been the source of the outbreak and poor sanitation and hygiene could have been the vehicles for the outbreak. To test these hypotheses, we carried out a case-control study from February 17-24, 2015.

### Case control study

We conducted a frequency matched case-control investigation where two controls were matched to a case on the basis of their age groups (2-5, > 5-15, > 15-25, > 25-39, > 39 years) and village of residence, in Rongo (Migori) and Ndhiwa (Homa Bay) sub-counties. For entry into the study, we defined cases as watery diarrhea (at least three motions in 24 hours) in a person ≥ 2 years who were residents of Rongo or Ndhiwa sub-counties between January 23, 2015 and February 25, 2015. The controls were defined as the absence of diarrhea in any person of the same age group and residence as the case between January 23, 2015 and February 25, 2015.

We calculated a minimum sample size of 156 that included 52 cases and 104 controls. The assumptions made during sample size calculation included 80% power of the study; 95% confidence intervals, desired Odds Ratio (OR) to be detected = 3.0; prevalence of handwashing before eating among controls of 49% [15] and correlation for exposure between matched cases and exposure = 0.2 [17]. Systematic random sampling was used to select the cases from a line list. The selected cases were then traced to their residences with the assistance of the area Community Health Worker (CHW). Where a household had more than one participant eligible for selection as a case, the participant with the earlier date of onset of acute watery diarrhea was selected.

Residences of the controls were randomly selected by spinning an empty water bottle while still at the home of a case. The direction of the bottle top was observed and the home in that direction and nearest to that of the case was selected for enrolment of a control. In cases where there were two homes very close to one another and the direction of the spinning bottle was inconclusive, we spun the bottle a second time. If the selected home did not have a participant meeting the enrolment criteria for a control, we moved to the right and selected the next home or continued moving to the right until we found a home with an eligible control. To select the second control, we moved back to the home of the case and spun the bottle again.

### Data collection

### Case control study

We used a standardized questionnaire to collect data focused on the two week period before the date of interview. The same questionnaire was administered to cases and controls through face to face interviews. We collected demographic information and asked questions on possible risk factors such as handwashing practices, water sources, treatment and storage of drinking water, sources of food, hygiene and sanitation practices and any ceremonies that had been attended. We recorded the coordinates of the cases’ residence and health facilities where they were treated using a Global Positioning System (GPS) machine. All the controls were interviewed within 24 hours of interviewing the cases.

### Data analysis

### Retrospective review of cholera outbreak line list

Data was entered, cleaned and analyzed using Microsoft Excel. We calculated proportions for categorical variables and means and medians for continuous variables. We calculated case fatality rates by dividing the number of deaths by the total number of cases and attack rates (AR) by dividing number of suspected cases by the projected populations for the year 2015.

### Case control study

We analyzed data using Epi Info 7. Odds ratios (ORs), 95% CI and p-values were calculated and factors with p-values ≤ 0.05 were considered statistically significant. Variables with p-value ≤ 0.2 were then entered into a multivariable logistic regression to determine the independent factors associated with the outbreak.

### Ethical considerations

Verbal consent was obtained from all participants before the interview. Parents or guardians of participants < 15 years granted consent on their behalf and accompanied them during the interview. No participant identifying information was associated with the reported data. This investigation was a response to a public health emergency and as such did not require approval by an institutional review board and therefore the investigation protocol was approved by the Ministry of Health.

## Results

### Descriptive epidemiology

**Figure 1:**
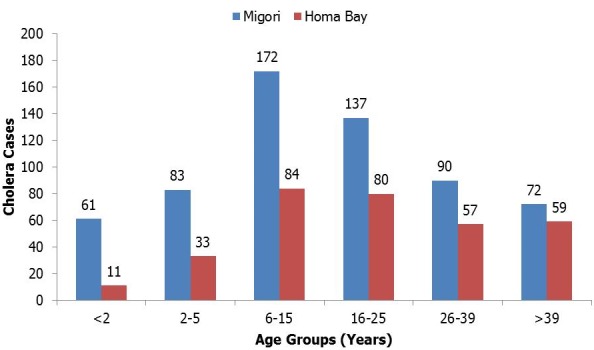
distribution of suspected cholera cases in Homa Bay and Migori by age-group, February, 2015

**Figure 2:**
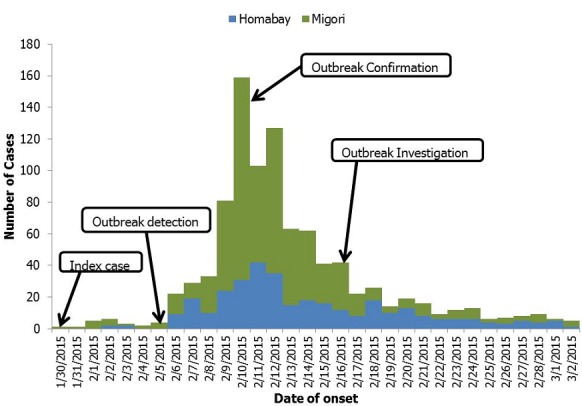
epidemic curve for the cholera disease outbreak in Migori and Homa Bay counties, Kenya, February, 2015

Between January 30, 2015 and March 2, 2015 there were 963 suspected cases; 38(4%) were confirmed cases. Females constituted 51% (486) of all the cases. Migori recorded 8 deaths (CFR = 1.3%) and Homa Bay recorded 4 deaths (CFR = 1.2%). Rongo sub-county reported six deaths (CFR = 1.0%) and had 595 (95%) of the cases in Migori with an attack rate (AR) of 495 per 100,000 persons while in Homa Bay, Ndhiwa sub-county reported 4 deaths (CFR = 1.3%) and had 93% (315) of the cases (AR = 156 per 100,000 persons) (Table 1). The ages of 939 (96%) cases were recorded and their median age was 16 years (Range < 1 to 100 years). The age group 6-15 years had the highest number of cases at 27% (256), while the least cases were in the age group < 2 years at 7% (72) (Figure 1). The median time period from onset of diarrhea to seeking treatment at a health facility was 0 days (Range 0 to 3 days). We recorded an exponential increase in the number of suspected cases with peaks on February 10 for Migori and February 11 for Homa Bay (Figure 2). Rectal swabs for culture were collected for 11% (105) of the suspected cases and V. Cholerae 01 serotype Ogawa was isolated from 18 (17%) samples. The same serotype was isolated from a sample of water from Riana River.

**Table 1:** distribution of cholera cases by sub-county of residence, Migori and Homa Bay counties, Kenya, february 2015

County	Sub county	No of Cases (%)^a^	Population	AR^b^ (per 100,000)
Homa Bay	Homa Bay town	15 (2)	111,068	14
	Ndhiwa	315 (33)	202,063	156
	Rangwe	9 (1)	117,038	8
	Total	339	430,169	79
Migori	Awendo	9 (1)	130,427	7
	Rongo	595 (62)	120,408	495
	Suna West	3 (<1)	112,720	3
	Nyatike	4 (<1)	173,193	2
	Uriri	13 (2)	138,616	9
	Total	624	675,364	92

a Percentage

b Attack Rate

### Case control

We recruited 52 cases and 104 controls. Females made up 61% (95) of all the participants. The age group > 5-15 had 33% (17) of enrolled cases and controls. Among the cases, 50% (26) had attained upper primary education while 22% (21) of the controls had attained upper primary education. Farming was the most reported occupation, practiced by 55% (29) of the cases and 59% (61) of the controls (Table 2). Fourteen (27%) of the cases reported having taken medication prior to visiting a health facility. Of these, four were on analgesics and 10 were on antibiotics. The symptoms reported by the cases varied; 69% (36) reported having suffered abdominal pain and fever while 12% (6) had blood-stained stools. In the health facilities, antibiotics were prescribed for 83% (43) of the cases; oral rehydration for 38% (63) and intravenous fluids for 33% (63). The median number of motions of loose stool in 24 hours among the cases was 5 (Range 1 to 20) while median duration between onset of diarrhea and seeking treatment was 1 day (Range 1 to 2 days). In addition, the median time period when the cases had diarrhea was 2 days (Range 1 to 7 days).

**Table 2:** distribution of cases and controls by socio-demographic factors, Migori and Homa Bay counties, Kenya, february 2015

Variable	Cases n^a^ (%)^b^	Controls n (%)
***Sex***		
Female	32 (62)	63 (61)
***Age in years***		
2-5	6 (12)	12 (12)
>5-15	17 (33)	34 (33)
>15-25	9 (17)	18 (17)
>25-39	11 (21)	22 (21)
>39	9 (17)	18 (17)
***Residence***		
Rongo	42 (79)	84 (81)
Ndhiwa	10 (21%)	20 (19%)
***Occupation***		
Salaried Worker	1(2)	1 (1)
Food Vendors	9 (17)	7 (7)
Farming	29 (55)	61 (59)
Unskilled Workers	13 (8)	35 (13)
***Level of Education***		
No Education	4 (8)	7 (7)
Lower Primary	12 (23)	12 (12)
Upper Primary	26 (50)	22 (21)
Secondary	10 (19)	63 (59)
***Religion***		
African traditional religion	1 (2)	6 (6)
Christian	50 (96)	98 (94)
Muslim	1 (2)	0

^a^Frequency, ^b^Percentage

On bivariate analysis, cases were more likely to use river Riana as their main source of drinking water (OR 2.32, CI = 1.17- 4.60) and were also more likely to lack a toilet or pit latrine in their homes (OR 2.28, CI = 1.15- 4.50). Cases were also more likely to practice food vending as an occupation compared to the controls (OR 2.9, CI = 1.01-8.30). Those who did not wash their hands after defecating were five times more likely to be cases (OR = 5.4, CI = 1.93-15.34), while those who practiced communal hand washing were more than four times likely to be cases (OR 4.7, CI = 1.93-11.40). Treatment of drinking water using any method was significantly protective for being a case (OR = 0.48, CI = 0.23-0.98). Those who chlorinated their drinking water and had chlorine available in the house were significantly protected from being a case (OR 0.17, CI = 0.04-0.72) (Table 3). On multivariate analysis, the independent factors associated with the outbreak were the practice of communal handwashing (aOR 6.5, CI = 2.30-18.11), lack of a toilet or pit latrine in the home (aOR 10.9, CI = 3.02-39.21) and food vending as an occupation (aOR 3.4, CI = 1.06-10.74) (Table 4).

**Table 3:** bivariate analysis of factors associated with outbreak of cholera in Migori and Homa Bay Counties, Kenya, February 2015

Factors	Cases n (%)	Controls n (%)	OR	95% CI	P-value
***Education***					
No Education	4 (8)	7 (7)	1.16	0.32-4.14	0.83
Lower Primary	12 (23)	12 (12)	2.3	0.95-5.56	0.06
Upper Primary	26 (50)	61 (57)	0.71	0.36-1.38	0.31
Secondary	10 (19)	22 (21)	0.88	0.39-2.05	0.78
***Occupation***					
Farming	29 (56)	61 (59)	0.88	0.45-1.74	0.73
Unskilled workers	13 (8)	35 (13)	0.65	0.31-1.39	0.27
Food vendors	9 (17)	7 (7)	2.9	1.01-8.30	0.04
Salaried Worker	1 (2)	1 (1)	2.02	0.12-32.9	0.62
***Water Exposure***					
Treating drinking water	32 (62)	80 (77)	0.48	0.23-0.98	0.04
Having Chlorine in the household	24 (46)	72 (69)	0.17	0.04-0.72	0.01
Drinking water outside home	27 (52)	36 (35)	2.04	1.04-4.02	0.04
Riana River as main drinking water source	24 (46)	71 (68)	2.32	1.17-4.60	< 0.01
Bathing in river	29 (56)	47 (45)	1.53	0.78-2.96	0.21
***Hygiene factors***					
Lack of toilet/pit latrine at home	29 (56)	37 (36)	2.28	1.15-4.50	0.02
Sharing latrine with a diarrhea patient	19 (37)	58 (56)	0.74	0.2-2.7	0.64
Lack of hand washing after defecation	13 (25)	6 (6)	5.4	1.93-15.34	<0.01
Hand washing after defecation without soap	5 (10)	8 (8)	1.7	0.51-5.41	0.4
Hand washing before eating without soap	11 (21)	9 (9)	2.9	1.1-7.5	0.03
Communal hand washing in basin	27 (52)	44 (31)	4.7	1.93-11.40	<0.01
***Food and fruits eaten***					
Mbuta	10 (19)	37 (36)	0.43	0.19-0.96	0.04
Omena	41 (79)	84 (81)	0.89	0.39-2.03	0.78
Porridge	50 (96)	93 (89)	2.96	0.63-13.8	0.15
Chicken	14 (27)	47 (45)	0.45	0.22-0.92	0.03
Ugali leftover	25 (48)	25 (24)	2.92	1.45-5.93	< 0.01
Sugarcane	45 (87)	84 (81)	1.53	0.60-3.89	0.37

**Table 4:** factors independently associated with cholera outbreak in Rongo and Ndhiwa, Kenya, February 2015

Factor	aOR	95% CI	P-Value
Communal hand washing	6.5	2.3-18.1	< 0.01
Lack of hand washing after defecation	10.9	3.0-39.2	< 0.01
Food vending as an occupation	3.4	1.1-10.7	0.03

aOR; Adjusted Odds ratio

## Discussion

We sought to identify the risk factors for the outbreak in Migori and Homa Bay counties. From our findings, improper handwashing was a risk factor for being a case. This included both communal handwashing in a basin with non-running water and washing hands without soap after defecation. Vending of food as an occupation was also a risk factor for the outbreak. Proper handwashing technique with water and soap, both before meals and after defecation is an intervention that was reported as a protective factor in a study in Zambia [18]. Proper handwashing reduces person-to-person transmission of cholera and ultimately reduces the impact of the outbreak as has been reported in several past studies [3,15,19,20]. Handwashing should be promoted during health promotion campaigns in the community as part of behavior change communication during outbreak situations. Vending of food was a risk factor in this outbreak, possibly because food vendors also consumed the food they were selling, that could have been contaminated. Although no specific food was identified as a risk factor in this study, contaminated food is a known risk factor, especially where the population at risk are able to access food from the food vendors [21-23].

Although no statistically significant association between treatment of water and being a case, the households that practiced in-house chlorination of drinking water and were able to provide sachets of chlorine during the interviews were less likely to have cases. Chlorination of water is a measure that is known to be protective against cholera infection during outbreaks [3,18,23]. This study area has been reported to have gaps in knowledge and practice of water treatment as a cholera preventive measure in a past survey [24] and this implies need for sustained health education concerning water treatment because habitual water treatment with chlorine and provision of safe water can be protective factors even in large outbreaks [3,9]. Although absence of pit latrine or toilet in a home is a known risk factor for cholera outbreaks, we did not find any association between absence of latrine in a home and being a case. Although there was clustering of cases around Riana river and *Vibrio cholera* was isolated from the river’s water, there was no association between using the river as main source of drinking water and being a case. Similar studies in Central African Republic and Tanzania also reported this finding [21,25].

This implies that there could have been other vehicles of transmission. This outbreak had a CFR of 1.3%, which was marginally higher than the WHO recommended CFR of < 1%. The MoH and partners in the two counties had put up several cholera treatment centres and distributed chlorine tablets to the households. In addition, patient follow up and tracing of close contacts was done and these significantly contributed to keeping the CFR lower compared to 2010 outbreak in Migori which had a CFR of 9% [16]. That study recommended early detection and confirmation of outbreaks in the area, a gap which we still observed in this study. Early detection and laboratory confirmation was observed to have resulted in reduced CFR in a similar study in Central African Republic [21]. Few serological tests were conducted because the laboratories in Migori and Homa Bay did not have the capacity to carry out these tests. The serotype was Cholerae 01 serotype Ogawa unlike the previous one in Migori county in 2010 which was caused by serotype Inaba [16] and the epidemic curve was suggestive of a common source outbreak, although in Homa Bay, multiple waves were observed which could signify person to person transmission, possibly due to gaps in detection of cases in the community and making a follow up to trace contacts of the cases. This implies need for setting up a prospective cholera surveillance system so as to enable early detection of outbreaks in future.

Our study has potential weaknesses. The interventions carried out by the counties included provision of chlorine sachets for water treatment at the household level; therefore there was a likelihood of the residents changing their practices with a resultant misclassification of their exposure status which could have considerably biased their risk of infection. *Vibrio cholera* was isolated from a small fraction of the cases and there was a possibility that non-cholera cases were included in the line list and this would have diluted the measure of effect. We also selected the controls from the same village of residence as the cases and they could have shared similar exposures as the case.

## Conclusion

There was multiplicity of factors associated with the outbreak, but inadequate hygiene and sanitation were the most likely causes of the outbreak. Health promotion should be provided to the local population on the need to construct and use latrines in their homes, plus correct handwashing procedures using soap and running water.

### What is known about this topic

- Cholera is an infectious disease that is endemic in most parts of sub Saharan Africa, including Kenya;
- Early detection and confirmation of cholera outbreaks leads to quick response and reduces the impact of the outbreak in the population;
- Risk factors for cholera include consumption of contaminated and untreated water and improper hand washing procedures following defecation.

### What this study adds

- We found that communal handwashing in a basin with non-running water, lack of handwashing with soap after defecation and being a food vendor were risk factors of being a case during cholera outbreak in Western Kenya.

## Competing interests

The authors declare no competing interest.
